# Pneumococcal Vaccines: Past Findings, Present Work, and Future Strategies

**DOI:** 10.3390/vaccines9111338

**Published:** 2021-11-17

**Authors:** Giuliana S. Oliveira, Maria Leonor S. Oliveira, Eliane N. Miyaji, Tasson C. Rodrigues

**Affiliations:** Laboratório de Bacteriologia, Instituto Butantan, São Paulo 05503-900, SP, Brazil; giuliana.oliveira@esib.butantan.gov.br (G.S.O.); tasson.rodrigues@esib.butantan.gov.br (T.C.R.)

**Keywords:** *Streptococcus pneumoniae*, pneumococcus, vaccine, PCV, PPV, whole cell, recombinant protein, new technologies

## Abstract

The importance of *Streptococcus pneumoniae* has been well established. These bacteria can colonize infants and adults without symptoms, but in some cases can spread, invade other tissues and cause disease with high morbidity and mortality. The development of pneumococcal conjugate vaccines (PCV) caused an enormous impact in invasive pneumococcal disease and protected unvaccinated people by herd effect. However, serotype replacement is a well-known phenomenon that has occurred after the introduction of the 7-valent pneumococcal conjugate vaccine (PCV7) and has also been reported for other PCVs. Therefore, it is possible that serotype replacement will continue to occur even with higher valence formulations, but the development of serotype-independent vaccines might overcome this problem. Alternative vaccines are under development in order to improve cost effectiveness, either using proteins or the pneumococcal whole cell. These approaches can be used as a stand-alone strategy or together with polysaccharide vaccines. Looking ahead, the next generation of pneumococcal vaccines can be impacted by the new technologies recently approved for human use, such as mRNA vaccines and viral vectors. In this paper, we will review the advantages and disadvantages of the addition of new polysaccharides in the current PCVs, mainly for low- and middle-income countries, and we will also address future perspectives.

## 1. Introduction

*Streptococcus pneumoniae* (Spn, the pneumococcus) is a frequent component of the human nasopharyngeal microbiota, but it is also an important pathogen, especially in children under five years of age and the elderly, as it is responsible for several hospitalizations and deaths [1,2]. Pneumococci cause several diseases ranging from sinusitis and otitis media to life-threatening diseases such as pneumonia, bacteremia and meningitis. The majority of the strains express a capsule, the most important virulence factor, which is associated with escape from opsonophagocytosis and other immune responses [3,4,5]. The capsule is composed of polysaccharides (PS) that can be chemically and immunologically classified into 100 serotypes [6]. Spn is genetically highly variable, especially in loci related to PS production and antibiotic resistance. In fact, Spn has mechanisms to rapidly adapt and evolve through recombination [7,8,9,10,11].

Lower respiratory tract infections (LTRI) have been the fourth leading cause of death in the world since 2000, but a reduction from 3.1 million in 2000 to 2.6 million in total deaths caused by LRTI was observed in 2019 [12]. Pneumonia cases alone were responsible for 15% of all deaths in children under 5 years of age [13]. The World Health Organization (WHO) denoted Spn as an important human pathogen, as it is responsible for more than 50% of all deaths caused by LTRI [2,13,14]. The WHO also established that vaccination is the most important policy to control pneumococcal disease [15].

The first pneumococcal vaccine dates back to 1909 in the United States, where a whole cell vaccine was first licensed [16]. Between 1923 and 1925, the characteristics of the PS, initially named soluble specific substance of pneumococcus (SSS), were described from some serotypes [17,18,19]. Based on that discovery, many groups started to work with pneumococcal PS as vaccines, even in the wartime period [20,21,22,23]. Additionally, during this time, two pneumococcal polysaccharide vaccines (PPV) with six different serotypes in two different formulations based on the prevalence of the serotypes in adults and infants, were licensed in the United States, but they were later removed from the market due to skepticism from physicians on the implementation of vaccines in clinics [24]. In the 1970s, new efforts for the development of pneumococcal vaccines were made, especially by Robert Austrian, who was responsible for cataloguing serotypes from pneumococcal diseases and leading clinical trials with 7-valent and 12-valent vaccines which later evolved to include 14 different serotypes. In 1983, some companies started to produce the 23-valent PPV (PPV23), composed of purified PS from 23 different serotypes, after a position of WHO supporting its use [16]. PPV23 induces a T-cell independent response, and for this reason, it is not indicated for children under two years. Even in people older than 60, the age group for which this vaccine is indicated, it presents a lower response when compared with young adults [25,26,27]. After 2000, another generation of vaccines became important: the pneumococcal conjugate vaccines (PCV). PCVs were developed by the conjugation of PS to carrier proteins, which rendered a T-cell-dependent response to PS with the induction of memory T-cells and protection of infants [28,29,30,31]. After the introduction of these vaccines, a huge decrease in pneumococcal disease and colonization was observed, as was reflected by the reduction of the circulation of vaccine serotypes and the protection of unvaccinated people through the herd effect [30,31,32,33,34]. On the other hand, in the period after introduction of PCVs in immunization programs, an increasing trend in the incidence rate of serotypes not included in the vaccines was observed. This phenomenon led to concerns regarding vaccine efficacy, especially when an increasing prevalence in serotypes more related to strains resistant to antibiotics was observed [34,35].

In this review, we aim to explore and exemplify the importance of pneumococcal vaccines and expose the necessity of exploring next generation strategies. We present here a variety of models and platforms to produce and design new vaccines to fight pneumococcal disease.

## 2. Inclusion of New Polysaccharide Serotypes into PCVs

Licensed PCVs have been extremely important in reducing pneumococcal disease worldwide. These vaccines included 7 to 13 PS from different pneumococcal serotypes conjugated to carrier proteins [36]. After the introduction of PCV7 in the immunization program of several countries, a high rate of protection of vaccinated children has been demonstrated, with reductions in cases of pneumonia and invasive pneumococcal disease (IPD) [37,38,39,40,41]. However, a few years after the introduction of these vaccines, serotype replacement was observed, with previously circulating serotypes included in the vaccine being substituted by non-vaccine serotypes [42,43,44,45].

Most countries have no indication for PCV use in young adults or the elderly, and some studies show a decline in cases of IPD caused by some vaccine serotypes in people over 65 years of age by herd immunity [43,46]. However, it is still difficult to clearly confirm the effect of herd immunity on community-acquired pneumonia (CAP) due to the routine use of bacterial isolation to determine pneumococcal serotypes [47]. More sensitive techniques, such as qPCR, would be required for a better evaluation. Regardless of indirect protective capacity, adults and the elderly are usually colonized and have pneumococcal disease caused by a wider variety of serotypes than children [48,49]. Therefore, current PCVs do not meet the specific needs of these populations.

Currently, new vaccines are being studied which aim at broader protection for serotypes not included in licensed PCVs. Vaccines with 11, 12, 15 and 20 serotypes are in advanced stages of clinical trials in humans, having already demonstrated safety and tolerability in addition to non-inferior induction of antibodies and opsonophagocytosis of pneumococci compared to results obtained with PCV10 and PCV13. Moreover, promising results for the new serotypes included were obtained for children, adults and elderly people [50,51,52,53,54,55,56,57]. In the pre-clinical phase, several other conjugate vaccines, with up to 30 serotypes, are under study [58,59].

In order to predict the formulation of new PCVs, it is possible to propose the inclusion of serotypes with the highest probability to increase their incidence in diseases according to PS characteristics. For example, capsule monosaccharides with lower carbon content or molecules with a pronounced negative charge are able to better evade the immune system [60]. However, several other variables can interfere with this prediction. For example, it is not possible to guarantee that new PCVs will have the same impact in serotype reduction and replacement as vaccines already in use in the population. For these reasons, new PCVs would need to constantly increase their valence, which leads to the question of if developing and using these vaccines is applicable in the long term.

The need for continuous increases in PCV valence also involves a problem with conjugation. The crucial issue of a PCV is the binding process of a PS to a carrier protein that can induce a more effective immune response than the PS alone [61]. The qualitative and quantitative increase of these molecules in the immunizing formulation can cause immune response interferences, both in the vaccine itself, and in other vaccines administered previously, jointly, and subsequently. These interferences can cause an imbalance in several arms of the immune response, so they must be readjusted to each new vaccine formulation. Therefore, the continuous addition of new PS in pneumococcal vaccines may be limited by potential interference in childhood vaccine schedules [62,63].

The most obvious difficulty about PCV manufacturing is the complexity and the high process cost. These issues lead to insufficient vaccination coverage in low- and middle-income countries (LMIC), such as African and Asian countries, which in 2009 accounted for 95% of pneumococcal deaths [1]. More than ten years after PCV licensure, sub-Saharan African countries still have the highest mortality rate in children under five years old, with the lowest decrease in trends of pneumonia, morbidity and mortality among all countries [64]. These data are also a direct consequence of vaccines based on Northern Hemisphere-prevalent serotypes that do not fully represent the needs of countries with a wider spectrum of serotypes causing disease, such as low-income countries (LIC) [65].

Simpler and cheaper new vaccine strategies based on more conserved antigens could provide serotype-independent protection. These vaccines would provide protection from the early stages of pneumococcal carriage to the stages of invasive disease through strategies that induce different immune responses.

## 3. New Perspectives in Classical Vaccines

A vaccine based on protein antigens would be able to protect against all Spn considering the selection of highly conserved proteins that must be present in most clinical isolates [66]. In addition, the manufacturing process should be simpler and cheaper than for PCV [67], providing broad access to populations from LMIC.

Their potential benefits depend on the ability to induce antibody production and cellular immune responses that will prevent carriage and disease [68,69]. The first step towards the selection of protein antigens is the extensive knowledge of the role of virulence factors of Spn [70]. The first virulence mechanism during the infection process is the reversible phase variation between transparent and opaque phenotypes, which occurs during carriage and invasion. The transparent phase predominates and seems to be required for carriage in the nasopharynx, where capsule expression is reduced to facilitate adhesion to epithelial cells, promoting colonization and expansion of the colonies. The expected response of protein vaccines is based on the recognition of the exposed molecules at this stage by antibodies and Th17 cells, leading to reduced colonization and, consequently, invasive diseases. It is also expected that they induce herd effect in the population [3,69,71,72]. The opaque phase predominates during invasion, with increases in capsule thickness promoted by higher production of PS. This mechanism protects bacteria from opsonophagocytosis by covering most of the epitopes and avoiding antibody attachment to the cell surface [73,74,75]. Protection against pneumococcal diseases is mainly mediated by high levels of antibodies and is also dependent on the recruitment of immune cells, especially neutrophils [76,77,78]. Some protein antigens can be recognized by the immune system independently of phase variation, and several have been considered as potential antigens for a new generation of vaccines:

- Pneumococcal histidine triad protein D (PhtD) is a highly conserved surface protein [79] involved in metal ion homeostasis, avoidance of complement deposition, adherence to host cells [80,81], and other virulence mechanisms in the lungs [82];
- Pneumolysin (Ply) is a cytolysin released by Spn during autolysis which leads to proliferation in the lungs and invasion of the bloodstream [81]. Ply induces formation of pores in cholesterol-rich membranes and activates the complement system, inducing a high inflammatory response [66], which may lead to lung injury and neuronal damage [80]. Its application in vaccines is proposed with its detoxified form (PdT) with the aim of inducing antibodies that neutralize Ply activity and inhibit its adhesion to epithelial tissue [81];
- Pneumococcal surface protein A (PspA) is a choline-binding protein responsible for inhibiting the activation and deposition of complement C3 component and for inhibiting apolactoferrin bactericidal activity [83,84]. It is a protein present in virtually all Spn isolates, and variations in their amino acid sequence classify it into six clades and three families [85]. Its N-terminal region is exposed on the capsule surface and exhibits a high degree of cross-reactivity [86,87];
- Pneumococcal choline-binding protein A (PcpA) is a surface protein with a role in adhesion, mainly to lung epithelial cells [88].

Recently, several proposals of protein-based vaccines have advanced from the pre-clinical phase to clinical trials in humans (Table 1). Here, we review strategies that have thus far undergone clinical trials, but it is important to remember that there are many other proteins that were tested only in animal models. A phase I study used a vaccine based on several pneumococcal surface antigens, including PspA, pilus proteins and PdT, obtained by purification from bacteria that were cultivated in conditions leading to upregulated expression of these proteins. This non-adjuvanted vaccine was considered safe in adults and demonstrated a significant increase in IgG titers against several Spn antigens. In addition, serum from immunized individuals was capable to neutralize Ply hemolytic activity in different serotypes [89].

The safety and tolerability of PdT and PhtD were evaluated in two separate studies with single-antigen formulations adjuvanted with aluminum hydroxide in adults. Both works showed promising results regarding the safety profile. PdT proved to be highly immunogenic, and serum from immunized individuals was able to neutralize Ply activity [66]. PhtD immunization was also safe and immunogenic; however, adverse reactions and induction of antibodies proved to be dose-dependent [67].

Other formulations containing two or three antigens have also been extensively tested in phase I clinical trials. The administration of PcpA + PhtD in two doses with three concentration levels for each protein, combined or not with aluminum hydroxide, were tested. All formulations proved to be safe with a considerable increase in antibody levels, which reached a plateau with the intermediate concentration of each protein. Furthermore, the addition of an adjuvant was not able to increase the immune response and was shown to be more reactogenic in adults [88]. Immune sera from volunteers were shown to protect mice in a passive immunization model against serotype 3 Spn (strains A66.1 and WU2) [79]. In another study, PdT + PhtD + PcpA was administered to adults, toddlers, and infants, with or without an adjuvant. All formulations were well tolerated in the three age groups and capable of inducing antibodies against all antigens. In infants, the target population of the vaccine, three doses with an adjuvant were necessary to obtain the best humoral response [90].

Another promising strategy is to complement PCVs with protein candidates (Table 1). This approach was performed using PdT and PhtD administered with PCV10 in phase I and II studies in adults, toddlers and infants with two doses and a booster dose. The formulation proved to be safe and effective, inducing an increase in the levels of anti-Ply and anti-PhtD antibodies and not negatively affecting the response to PS [81,82,91]. These proteins were also co-administered with DTPa-HBV-IPV/Hib (Infanrix Hexa, GSK), a vaccine commonly present in childhood vaccination schedules, and no differences were observed in the immune response for this vaccine [91]. However, this formulation did not lead to a reduction in nasopharyngeal carriage in infants in The Gambia [92]. In another study, PdT and PhtD were co-administered with PCV13 in Native American infants, generating a robust antibody response against the proteins used. However, efficacy against acute otitis media and acute LRTI was not verified in this population [93].

PdT and PhtD were also used as PS carrier proteins in a phase I study with an elderly population. For the 8 polysaccharides included in the formulation, only 2 (19A and 22F) were conjugated to pneumococcal proteins. The results demonstrated the vaccine safety in this age group and the ability to increase antibodies against the proteins. However, the antibody induction was greater in the population immunized with the proteins alone. Regarding the response to 19A and 22F conjugates, both were shown to be immunogenic, with increased opsonophagocytic activity after two doses when compared to the PPV23, indicating that PhtD and PdT can improve the response against pneumococcus [80].

An alternative to purified protein vaccines is live-vector based vaccines (Table 1), such as the one that underwent a phase I study where live-attenuated *Salmonella typhi* strains were used as vectors for oral PspA delivery in adults. Immunization was shown to be safe and well tolerated; however, these vaccines were not able to induce anti-PspA antibodies, probably due to pre-existing antibodies against the vector in participants [68].

A second alternative is the classic whole cell vaccine (Table 1), which presents a wide variety of antigens in their native form, is self-adjuvanted by presenting toll-like receptor agonist molecules, and is a good choice of vaccine for the immunization of children in LIC due to its low manufacturing cost [69,94,95]. It is expected that in countries with a high rate of colonization in early childhood, these vaccines could reduce pneumococcal colonization in the nasopharynx, maintaining the bacterial density at a minimum [69] and preventing the spread of new serotypes.

The first human trial was carried out to verify the safety, tolerability, and immunogenicity of a whole cell vaccine from a non-encapsulated strain, adsorbed on aluminum hydroxide and administered in three doses in adults. The results demonstrated that the vaccine was well tolerated and induced B and T cell responses as well as antibodies against Ply and PspA in 75% of the participants who received the highest dose used in the study. Furthermore, the antibodies demonstrated a protective capacity through the neutralization of Ply toxicity and by passive immunization of mice challenged with Spn serotype 3 (A66.1) [95].

The generalization of results obtained from clinical trials should be done with caution, as differences between populations can lead to different vaccine efficacy results. In LMIC, differences in nutrition and basic sanitary and living conditions can modify the response to vaccines [69,81], as in the case of children from The Gambia, who present with pneumococcal carriage since early infancy, a situation that does not occur in European children [92]. In the elderly, immunosenescence is a widely known condition that reduces vaccine effectiveness in this population [80,96].

New generations of vaccines that are not based on PS will have important regulatory barriers to overcome, as the ideal endpoints and correlates of protection for these studies have not yet been defined [97]. These new vaccines may not have their protection based on antibodies that induce opsonophagocytosis, such as PCV, but on several other immune responses that would also be able to reduce carriage and/or pneumococcal disease [69].

**Table 1:** Pneumococcal vaccines: vaccines licensed and in clinical trial.

Vaccine	Type	Adjuvant	Manufacturer	Clinical Trial Fase
PPV23 [98]	PS	None	Merck ^1^	4 (licensed)
Synflorix (PCV10) [99]	Conjugated PS	Aluminum phosphate	GSK ^2^	4 (licensed)
Prevnar 13 (PCV13) [100]	Conjugated PS	Aluminum phosphate	Pfizer ^3^	4 (licensed)
Ply (PlyD1) [66]	Recombinant protein	Aluminum hydroxide	Sanofi Pasteur ^4^	1
PhtD [67]	Recombinant protein	Aluminum hydroxide	Sanofi Pasteur ^4^	1
PhtD [96]	Recombinant protein	None, aluminum phosphate or AS02V	GSK ^2^	1/2
PcpA + PhtD [79,88]	Recombinant protein	Aluminum hydroxide	Sanofi Pasteur ^4^	1
Ply + PcpA + PhtD [90]	Recombinant protein	None or aluminum hydroxide	Sanofi Pasteur ^4^	1
Ply + PhtD PCV10/PCV13 [81,82,91,92,93,97]	Recombinant protein administered with PCV10 or PCV13	None or aluminum phosphate	GSK ^2^	1/2
Ply + PhtD PCV8 [80]	Recombinant protein as PS carrier	AS02V or aluminum phosphate	GSK ^2^	1
*Salmonella Typhi* expressing PspA [68]	Live vector	None	Arizona State University/Saint Louis University	1
PnuBioVax [89]	Subunit inactivated	None	ImmunoBiology Ltd.	1
wSp [95]	Inactivated whole cell	Aluminum hydroxide	PATH	1

^1^ Merck Sharp & Dohme Corp; ^2^ GlaxoSmithKline plc; ^3^ Pfizer Inc.; ^4^ Sanofi-Aventis Group.

## 4. Future Strategies for Vaccine Development

In 2019, the world observed the spread and the magnification of SARS-CoV-2 epidemic. In response to that, many groups started to use technologies they have been working on for years to fight the virus. Some first in-human approved technologies got more attention, such as the mRNA-based vaccines that received the Emergency Use Listing between December 2020 and April 2021, but there are several other candidates at different stages of clinical trials [101,102]. Additionally, in this period, vaccines based on non-replicating viral vectors containing recombinant DNA received the same approval, becoming another important option for vaccine technology [103]. These important advancements brought us new opportunities to facilitate development and approval of new vaccines against different pathogens. Emergency use of vaccines based on these new technologies will certainly impact the field of vaccine development against several pathogens, including pneumococci. Protective responses against viral and bacterial pathogens usually differ, but since most vaccines approved against SARS-CoV-2 rely on the induction of antibodies, such technologies may be effective against extracellular bacteria as well.

Thinking about the development process of mRNA vaccines, the mRNA by itself has low efficiency in inducing protection, but combined with other techniques, such as polymer- or lipid-based nanoparticles, this kind of formulation can have a 60 to 95% efficiency in transfection rates, with a peak of protein production around 5 h after the administration [104]. Depending on the composition and route of administration, the potential of expression of antigens at sites different from the inoculation can also lead to a broad and long-lasting protection against diseases, especially in early stages of infection in the mucosa, with a rapid response against the pathogen [105,106,107,108]. Data have indicated that there is a similar immunological response induced by mRNA vaccines in both young and older adults, and recent trials have further confirmed that an identical response occurs in adolescents [109,110,111,112]. In recent clinical trials, this kind of vaccine has shown the best effectiveness when compared to other vaccines used against COVID-19, with a high antibody titer and stimulation of a cellular response polarized to Th1 [113,114]. The induced immune response can also be improved by the employment of an amplifying RNA sequence, which has a promoter sequence in the same molecule (self-amplifying RNA) or in another molecule at the same site (trans-amplifying RNA) that is responsible for producing more RNA [115]. Another interesting point of mRNA vaccines is their easy exchangeable application, exemplified by the announcement by Moderna TX of a phase 1 clinical trial with two HIV vaccine candidates based on the successfully developed platform (ClinicalTrials.gov Identifier: NCT05001373) [116]. Other groups are also working on development of mRNA-based vaccines against HIV, influenza, Zika, chikungunya, and other pathogens with promising results [108,117,118,119].

At the same level of importance of the mRNA application, nanotechnology applied to protein antigens has been explored in vaccine development for a long time against several pathogens, including pneumococci. The variety of components used to produce nanoparticles (NP) is enormous, from biodegradable polymers and lipids to inorganic materials, such as gold and silver NP [120,121]. The production of NPs has also many platforms to work with: emulsions with natural or synthetic molecules, such as polymers and lipids; self-assembling proteins particles (SAPP), which are protein monomers bound together to form blocs at the nanoscale size; virus-like particles (VLP), which are capsid proteins folded into a particle but without any genetic material; and inorganic NPs. Some of these particles have been approved as therapeutic drugs for human use [120,122], but the first vaccine using nanotechnology received its authorization during the COVID-19 pandemic. This list can increase in upcoming months, with at least two other vaccines based on NPs with recombinant proteins and five others based on VLP in different stages of clinical trials [102]. There are many examples of nanovaccines that have been applied against several pathogens, such as human papillomavirus, Ebola, influenza, Leishmania, *Streptococcus pyogenes*, *Mycobacterium tuberculosis*, *Streptococcus agalactiae*, and many others [120,123,124,125,126,127,128,129,130,131].

In fact, a good effort has been applied to develop next-generation pneumococcal vaccines based on many platforms, including NPs [132,133]. In one study, the process of a mineralized Ply fused with the same protein without the inorganic component, forming a calcium phosphate NP, substantially increased protein thermal stability and resistance to proteases [134,135]. Nanotechnology formulation also makes it possible to work with more than one antigen. Recently, a cationic cholesteryl pullulan nanogel encapsulating three PspAs from clades 1, 2 and 3 was shown to induce a high antibody titer with the induction of complement deposition on the surface of the bacteria; it was also shown to protect against pneumococci in an animal challenge model [136]. Another work showed that a NP synthesized with sorbitol diacrylate and polyethyleneimine adsorbed with PspA from clade 2 induced specific antibodies in serum and in the lungs, and this protection was highly efficient and long-lasting against bacteria in a lethal challenge model. They also proved that this NP leads to a Th2 response and the induction of protection is related to the interaction of dendritic cells and T cells [137]. Another group showed that the encapsulation of PspA from clade 2 in NP made with 1,8-bis-(p-carboxyphenoxy)-3,6-dioxaoctane (CPTEG) copolymerized with 1,6-bis-(p-carboxyphenoxy)hexane (CPH) or CPH copolymerized with sebacic acid (SA) induced an antibody response and protection in a lethal challenge model, even after reduction of the administered dose. This formulation also preserved the antigen characteristics, even when stored at room temperature [138]. It was also shown that NP synthesized with the copolymer poly(glycerol adipate-co-ω-pentadecalactone) (PGA-co-PDL) adsorbed with PspA from clade 4 induced an antibody response with some cross-reaction with other clades as well as partial protection against pneumococci in a lethal challenge [139,140]. The possibility to work with in silico strategies can also increase the efficiency of these formulations; one example could be the development of an SAPP designed to be better recognized by the immune system by using the sequences of four different antigens [141].

Other technologies have also received attention after the COVID-19 pandemic, such as non-replicating viral vector vaccines and DNA vaccines. The first one is already being used in the population, and the second one is undergoing several clinical trials [101,102,142]. The diversity of vectors to be applied in vaccine development is enormous and, in fact, at least 22 COVID-19 vaccine candidates currently in clinical trials are based on this approach [102]. This kind of platform has been studied for a long time against several pathogens, and during the COVID-19 pandemic, it has been shown to be safe and capable of inducing a good protection, despite some adverse reactions in some cases [143,144,145,146,147]. Among the COVID-19 candidate vaccine clinical trials, 10 of them are based on DNA vaccine technology. Although some works have indicated this technology as an opportunity to fight pneumococcal disease, there are no recent publications on that [148,149,150,151,152].

Recently, another possibility to produce vaccines has been raising attention, and it is based on outer membrane vesicles (OMV). These particles are naturally formed in Gram-negative bacteria and are composed mainly by LPS, an outer membrane, periplasmic proteins, and phospholipids. It is also possible to induce the formation of OMVs through sonication or detergent treatment [153]. Their application as vaccines can be improved by genetic modifications to increase OMV production and protein expression, inducing heterologous protection in animals [154]. In fact, OMVs from *Escherichia coli* presenting glycans with similarity to PS from serotype 14 were already studied and found to induce similar levels of antibodies and protection compared to PCV13 [155]. Another work showed that OMV from *Salmonella typhimurium* expressing PspA and PdT from pneumococcal strain TIGR4 were capable of inducing high levels of IgG and protecting against colonization [156]. Gram-positive bacteria were also shown to produce vesicles, specifically extracellular vesicles (EV). EV formation has also been described for Spn, and EV from ATCCBAA255 Spn strain was shown to be rapidly engulfed by mammalian cells, including immune cells, and to modulate cytokine responses [157]. Furthermore, immunization with EVs was shown to induce protection against the homologous as well as against a heterologous pneumococcal strain in mice [158].

## 5. Conclusions

Pneumococcal vaccines licensed up to the present time have greatly impacted human health and their importance is unquestionable. However, this impact also induced changes in bacterial population through the adaptation of Spn by selective pressure, leading to serotype replacement. This fact, combined with the constant increase of multidrug resistant bacteria, has raised Spn to the status of a major public health problem. It is imperative that new vaccines which are effective against a broad spectrum of serotypes and affordable to every country are developed. There are some possibilities for development on the horizon, ranging from protein-based and whole-cell vaccines to mRNA and viral vector vaccines that had their paths opened up by the COVID-19 pandemic. Therefore, it is possible to expect a new generation of pneumococcal vaccines based on some of the technologies presented here in the future.
